# The snowball effect of RNA binding protein dysfunction in amyotrophic lateral sclerosis

**DOI:** 10.1093/brain/awy091

**Published:** 2018-04-23

**Authors:** Pietro Fratta, Adrian M Isaacs

**Affiliations:** 1 UCL Institute of Neurology, and MRC Centre for Neuromuscular Disease, Queen Square, London, WC1N 3BG, UK; 2 Department of Neurodegenerative Disease, UCL Institute of Neurology, Queen Square London, WC1N 3BG, UK; 3 UK Dementia Research Institute at UCL, UCL Institute of Neurology, Queen Square London, WC1N 3BG, UK

## Abstract

This scientific commentary refers to ‘TDP-43 regulates the alternative splicing of hnRNP A1 to yield an aggregation-prone variant in amyotrophic lateral sclerosis’, by Deshaies *et al*. (doi:10.1093/brain/awy062).

This scientific commentary refers to ‘TDP-43 regulates the alternative splicing of hnRNP A1 to yield an aggregation-prone variant in amyotrophic lateral sclerosis’, by Deshaies *et al*. (doi:10.1093/brain/awy062).

Amyotrophic lateral sclerosis (ALS) and frontotemporal dementia (FTD) are two degenerative disorders with a strong clinical overlap; no effective cure is available for either (Taylor *et al.*, 2016). Both disorders share the same hallmark pathology: the RNA binding protein (RBP) TDP-43, normally largely nuclear, is depleted from the nuclei of affected neurons and present in cytoplasmic inclusions. This occurs in nearly all ALS cases and in ∼50% of FTD cases. The identification of mutations in the TDP-43 gene, *TARDBP*, as causative for ALS provides further support for TDP-43 as a central player in disease pathogenesis, rather than a bystander. TDP-43 is involved in numerous RNA processing mechanisms including splicing. Over the last decade, ALS-causative mutations have also been found in other RBP-encoding genes, including *FUS*, *MATR3*, *HNRNPA1*, *SFPQ* and *TIA1*, sparking an interest in investigating RNA metabolism defects in ALS (Taylor *et al.*, 2016). Although the role of TDP-43 in RNA metabolism is well defined, a number of questions remain, including (i) whether alterations in RNA functions play a role in disease; and (ii) whether the heterogeneous ribonucleoproteins (hnRNPs) implicated in ALS act independently or co-operatively. In this issue of *Brain*, Deshaies and co-workers help address these questions by showing that TDP-43 pathology induces expression and pathological changes in another ALS-hnRNP, hnRNP A1 (Deshaies *et al*., 2018).

Deshaies *et al*. show that TDP-43 depletion upregulates the transcript and protein levels of two distinct *HNRNPA1* isoforms; the well characterized hnRNP A1 isoform that excludes exon 7B, and a previously poorly characterized isoform, hnRNP A1B, which includes exon 7B. Inclusion of exon 7B extends the low complexity domain (LCD), which has been implicated in aggregation of hnRNPs (Kim *et al.*, 2013). Accordingly, the increase in *HNRNPA1B* upon TDP-43 nuclear depletion contributes to increased aggregate formation and toxicity.

Recent work has shown that TDP-43 aggregation within cells leads to a loss of the normal nuclear splicing function of TDP-43 (Mihevc *et al.*, 2016). In addition, an important aspect of TDP-43 biology is the ability of TDP-43 to bind to and reduce its own transcript levels, termed autoregulation (Ayala *et al*., 2011). TDP-43 nuclear depletion in post-mortem brains is associated with a loss of *TARDBP* autoregulation, indicating that loss of TDP-43 function occurs in the brains of patients (Koyama *et al.*, 2016).

Deshaies *et al*. propose that TDP-43 regulates *HNRNPA1* by altering its splicing, thereby linking loss of splicing function to a toxic mechanism with a potential role in pathogenesis. However, the authors show too that TDP-43 depletion increases *HNRNPA1* promoter activity, which could also explain the increases in transcript and protein levels. Therefore, while the potential role of TDP-43 in exon 7B splicing is intriguing, it will need support from further studies to be confirmed. Indeed, although the authors report predicted TDP-43 RNA binding sites in introns flanking exon 7B, these are not present in current publicly available CLIP (Tollervey *et al.*, 2011) and ENCODE CLIP datasets, which map the genome-wide RNA-binding sites of TDP-43. Strong binding sites are present in *HNRNPA1* exon 8 and 3’UTR, and the ENCODE CLIP data also show additional binding sites in exon 7; as the minigene used to investigate the relationship between TDP-43 binding and exon 7B splicing does not contain exon 8, further studies using endogenous *HNRNPA1* will be important to investigate the potential contribution of these sites to exon 7B regulation. In publicly available ENCODE RNA-seq datasets for cellular TDP-43 knock-down, no noticeable increase in exon 7B inclusion occurs. This may indicate that TDP-43-mediated regulation of this splicing event is cell type-specific, or that other RBPs also play a role, and further experiments on different cell types may help address whether this could be a CNS-specific effect.

Glossary

**CLIP (cross-linking immunoprecipitation):** A technique that uses an antibody against a specific RNA binding protein (RBP) to pull down RBP-RNA complexes, followed by high-throughput sequence analysis to analyse and precisely locate RBP binding sites on RNA.

**ENCODE (the encyclopedia of DNA elements)**: An international consortium that is aiming to map all the functional elements in the human genome by using a wide range of experimental approaches.

**LCD (low complexity domain)**: Protein stretches containing low amino acid sequence complexity. These are very common in hnRNPs and play a crucial role in their phase separation properties and in the assembly of stress granules.

**Stress granules:** Cytoplasmic membrane-less bodies where, upon a variety of stress stimuli, mRNAs and proteins rapidly assemble. Translation is stalled in these granules, which disassemble after stress recovery.

**Figure 1:**
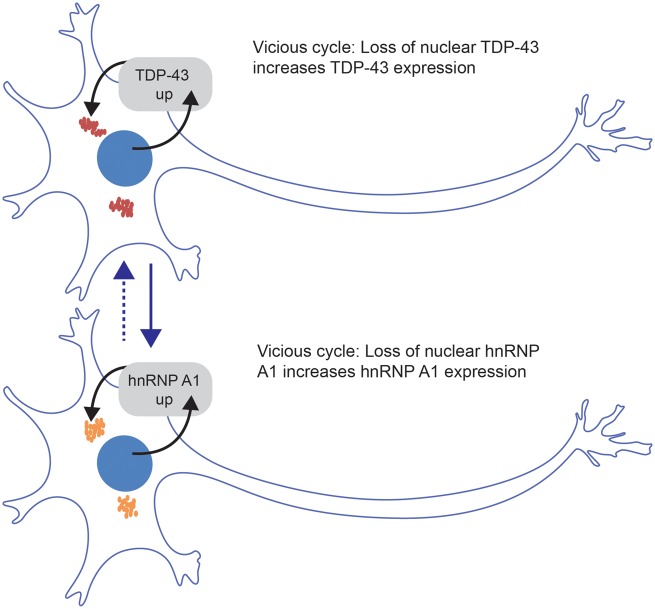
**ALS motor neurons are characterized by TDP-43 cytoplasmic inclusions and TDP-43 nuclear depletion, creating a vicious cycle where nuclear TDP-43 autoregulation at the mRNA level is lost, leading to increased TDP-43 levels.** Deshaies *et al*. show that this also leads to increased *HNRNPA1* expression and pathology, potentially initiating an *HNRNPA1* vicious cycle, which may in turn further affect TDP-43 function.

TDP-43 and hnRNP A1, like many other hnRNPs, are very tightly autoregulated through the binding of their own RNA in the nucleus, most likely because their functions are extremely dosage sensitive (Ayala *et al.*, 2011; Suzuki and Matsuoka, 2017). When cytoplasmic aggregates deplete the nuclear pool of TDP-43, *TARDBP* levels are upregulated, due to loss of autoregulation (Koyama *et al.*, 2016), leading to a vicious cycle of increasing RNA expression and cytoplasmic aggregation. Deshaies *et al*. show that TDP-43 loss of function leads to the hnRNP A1 protein becoming more cytoplasmic, with a concomitant reduction in nuclear levels in neurons from patients. It is intriguing to speculate that this may lead to an imbalance in *HNRNPA1* autoregulation, thereby triggering the onset of a parallel *HNRNPA1* vicious cycle (Fig. 1). TDP-43 and hnRNP A1 are known to interact and to act co-operatively in splicing events (D’Ambrogio *et al.*, 2009). The reduction in both proteins in the nucleus may therefore further enhance specific splicing alterations in ALS.

The manuscript by Deshaies *et al*. focuses on the link between TDP-43 and hnRNP A1, and highlights the fact that we do not know to what extent TDP-43 can influence other hnRNPs—an important question for further study. Recent reports suggest that an increase in levels of certain hnRNPs can rescue TDP-43 toxicity (Suzuki *et al.*, 2015; Appocher *et al.*, 2017). Therefore, whilst on the one hand we learn from Deshaies *et al*. that TDP-43 changes can cause alterations in hnRNPs likely contributing to disease, an increase in hnRNP levels can, conversely, rescue TDP-43 toxicity. The mechanism by which hnRNPs rescue TDP-43 toxicity and whether such approaches can reverse the vicious cycles discussed above need further study, but the results clearly emphasize the complex interplay between hnRNPs in FTD and ALS.

Accumulation of RBPs in ALS is typically seen in cases with mutations in these RBPs. Findings in non-mutant cases are less conclusive, although Matrin 3 accumulation has recently been described in sporadic ALS (Tada *et al.*, 2018). Another important finding reported by Deshaies *et al*. is that of hnRNP A1 cytoplasmic accumulations in ALS cases not carrying *HNRNPA1* mutations. These accumulations appear in the same neurons where TDP-43 inclusions and nuclear depletion occur, consistent with the proposed role for TDP-43 loss in their formation, but intriguingly they do not co-localize with TDP-43 inclusions. Quantification of hnRNP A1B aggregates in different sporadic and familial FTD and ALS subtypes would help further confirm the relevance of this pathway to other forms of FTD and ALS. Reduction of hnRNP A1 in nuclei in the absence of cytoplasmic inclusions has been described previously (Honda *et al.*, 2015). The generation by Deshaies *et al*. of an hnRNP A1B-specific antibody may help to clarify how much cytoplasmic aggregation is driven specifically by the hnRNP A1B isoform.

TDP-43, hnRNP A1 and the majority of RBPs involved in ALS share the presence of an LCD, which is important for their localization to stress granules, membrane-less organelles that assemble in the cytoplasm under stress conditions and which have been widely implicated in ALS pathogenesis. Indeed, the hallmark ALS inclusions have been proposed to derive from stress granules. The fact that hnRNP A1 and TDP-43 co-localize in stress granules, whereas they do not appear to do so in inclusions in patient neurons, is still compatible with this hypothesis. However, it may highlight a specific additional post-stress granule phase where aggregates of predominantly one RBP species are formed. The work of Deshaies *et al*. sheds new light on the hitherto less well studied hnRNP A1B isoform, which has an enlarged LCD that enhances aggregation and toxicity. This fits well with the hypothesis that LCDs contribute to the pathogenic fibrillization of RBPs involved in ALS (Kim *et al.*, 2013; Taylor *et al.*, 2016), thus providing new insights into the potential mechanisms by which hnRNP A1 can initiate disease.
